# Current anthelmintic treatment is not always effective at controlling strongylid infections in German alpaca herds

**DOI:** 10.1186/s13071-019-3588-3

**Published:** 2019-07-02

**Authors:** Luise Kultscher, Barbara Hinney, Ronald Schmäschke, Anja Joachim, Thomas Wittek

**Affiliations:** 1 University Clinic for Ruminants, Department for Farm Animals and Veterinary Public Health, University of Veterinary Medicine Vienna, Veterinärplatz 1, 1210 Wien, Austria; 2 Institute of Parasitology, Department of Pathobiology, University of Veterinary Medicine Vienna, Veterinärplatz 1, 1210 Wien, Austria; 3 Institute of Parasitology, Faculty of Veterinary Medicine, University of Leipzig, An den Tierkliniken 35, 04103 Leipzig, Germany

**Keywords:** South American camelids, Efficacy, Nematodes, *Haemonchus contortus*, Fenbendazole, Moxidectin, Monepantel

## Abstract

**Background:**

Endoparasites are considered a major health problem of South American camelids as shown in a recent survey among German and Austrian camelid owners. Although prophylactic and therapeutic measures such as application of anthelmintics are commonly used, treatment efficacy is usually not assessed. Owners have expressed significant concerns regarding the effect of antiparasitic therapy, so this study aimed to evaluate the outcome of anthelmintic treatment in German alpaca herds with different drugs.

**Results:**

Overall, 617 samples from 538 clinically healthy alpacas > 1 year-old from 27 farms (*n* = 11–157 animals/herd) were examined. The most common parasites detected by flotation were *Eimeria* spp. (75.1%) followed by strongylids (55.0%), *Nematodirus* spp. (19.3%), cestodes (3.1%) and *Trichuris* (2.7%). After initial coproscopical examination by flotation and strongylid egg quantification by the McMaster technique, positive animals excreting at least 150 eggs per gram of faeces were included in a faecal egg count reduction test (FECRT) using fenbendazole (*n* = 71 samples), moxidectin (*n* = 71) or monepantel (*n* = 66). Pre-treatment larval cultures (*n* = 23 positive pooled farm samples) revealed *Haemonchus* (87% of the farms), *Cooperia* (43.5%), *Trichostrongylus* (21.7%), *Ostertagia* (13.0%), *Nematodirus* and *Oesophagostomum* (4.3% each). Fenbendazole treatment reduced egg excretion by 45%, moxidectin by 91% and monepantel by 96%. On the farm level, 13/18 farms that used fenbendazole, 6/6 farms that used moxidectin and 2/5 farms that used monepantel had individual FECR values < 90% (fenbendazole) or < 95% (moxidectin, monepantel). *Haemonchus* and *Cooperia* were overrepresented on the farms with reduced treatment efficacy.

**Conclusions:**

Gastrointestinal strongylids are common in German alpacas and fenbendazole in particular was not sufficiently effective to reduce strongylid egg excretion. Although the FECRT could not unambiguously determine anthelmintic resistance in the present study, the finding that small ruminant strongylids, especially *Haemonchus*, are common in alpacas indicates that determination of effective anthelmintic doses, monitoring of efficacy and adapted (selective) treatment regimens must be implemented as part of sustainable deworming practices in this species in accordance with recommendations for ruminants.

## Background

South American camelids are becoming increasingly popular outside their native habitat [1–3]. In Germany, an estimated 20,000 alpacas are kept for a variety of purposes, including wool production (77%) and trekking (29%), and less frequently for animal-assisted therapy (12%), landscape management (3%) or simply as pets [4].

So far, only few published data are available regarding the endoparasites of these animals in their new environment and the pharmacological control options [5, 6]. In central Europe, information on camelid endoparasites is available for southern and central Germany [7–9], and, as a more comprehensive study, for Austria [10], all demonstrating a range of endoparasites, especially coccidia and strongylid nematodes, with variable but often high prevalence rates. Recently, a survey questioning owners of alpacas in Germany and Austria showed that endoparasites were regarded as a major problem in the herds and are diagnosed frequently (78.6% herd prevalence for gastrointestinal nematodes and 73.3% for coccidia; [4]). Prophylactic and therapeutic measures (including anthelmintic treatment with moxidectin as the most commonly used drug, followed by fenbendazole and monepantel, and consecutive treatment with different anthelmintics) are frequently applied. However, the effect of the treatment is not evaluated systematically (e.g. by post-treatment faecal examinations) and owners have expressed considerable concern about poor treatment efficacy, albeit without reliable data to support this view [4]. Larger farms in particular have reported cases of fatalities due to endoparasites of small ruminants, most commonly infections with *Haemonchus contortus*, the Barber’s pole worm [4].

After the first case report of ivermectin resistance of *H. contortus* in an alpaca herd in Australia [11], a recent systematic study described resistance to several anthelmintics (ivermectin, moxidectin, fenbendazole, closantel) in strongylids of Australian alpacas [12]. The large Australian sheep population may have contributed to the transfer of resistant nematodes to alpacas [12] since cross-transmission of a number of strongylid species (including *H. contortus*) between these hosts is common [13]. In Europe, resistance to doramectin has been reported on a Belgian alpaca farm and confirmed in a controlled field trial, and the main strongylid species involved was *H. contortus* [14].

We wanted to obtain an overview on the prevalence of endoparasites in the alpaca population in Germany and possible geographical variations, and determine whether the anthelmintic treatment commonly applied in the investigated herds is sufficient to control strongylid nematodes. For this purpose we conducted a follow-up study based on the previous questionnaire survey where German alpaca owners were asked to participate in faecal examinations of their animals [4]. Based on the survey results we hypothesized that commonly applied anthelmintics showed a decreased efficacy in alpacas in Germany. It was also assumed that *H. contortus* is common and plays an important role in the alpaca population.

## Methods

### Selection of animals and farms

The selection of the participating farms was based on a questionnaire on endoparasitoses [4] which provided general information on herd size, management, feeding, housing, environmental conditions, hygiene and the purpose of the animals. Specific veterinary aspects of the survey included results of previous faecal examinations, frequently diagnosed endoparasites, the owners’ perception of the current status of anthelmintic efficacy, deworming management and diseases and losses caused by endoparasitoses [4].

In this questionnaire, the breeders were also asked if they would be willing to contribute to a study exploring the efficacy of commonly used antiparasitic drugs. A further selection criterion for the participating farms was the anthelmintic drug predominately used in the herd. Since the study aimed to test the efficacy of three different drug groups (benzimidazoles, macrocyclic lactones and aminoacetonitriles), approximately equal numbers of farms using these drug classes were enrolled in the study.

The enrolled alpacas were older than one year, clinically healthy, of different sexes and had not been treated with anthelmintics for at least 12 weeks before the first sampling. Only medium-sized (11–50 animals) and large-sized herds (51–157 animals) were examined. From each farm, inclusion of at least 20 animals was intended; if this was not possible, repeated sampling was undertaken.

The final selection of the included farms was also based on consideration of the geographical distribution aiming to include farms from different regions of Germany (Table 1).

**Table 1:** Geographical distribution of farms and samples included in this study

	No. of farms	No. of samples (no. of samples examined by flotation)
North-west	9	200 (196)
Schleswig-Holstein	1	16
Lower Saxony	3	55
North-Rhine Westphalia	5	129
South-west	6	130 (125)
Hesse	2	33
Baden-Wuerttemberg	4	97
North-east	9	222 (201)
Mecklenburg-Vorpommern	1	14
Saxony	6	166
Thuringia	2	42
South-east	3	65 (65)
Bavaria	3	65
Total	27	617 (587)

### Faecal examinations

Individual faecal samples were collected at the beginning of the study and transferred to the laboratory on the same day. Faecal consistency was scored from 1 (physiological) to 4 (semi-liquid). Upon arrival, faeces were examined for the presence of parasitic stages by a combined sedimentation-flotation method. A walnut-sized piece of faeces was homogenized with water, sieved and transferred to a centrifugation tube. After centrifugation at 690×*g* for 8 min, the supernatant was discarded and the faecal pellet was re-suspended in approximately 12 ml of saturated sodium chloride solution (specific gravity: 1.18) and centrifuged again as above. Four to five drops were then removed from the surface of the suspension with a loop wire an examined under the microscope (100× magnification) for the presence of parasite stages. Helminth eggs were recorded qualitatively and oocysts of coccidian semi-quantitatively from 1 (very low, 1–3 oocysts/sample) to 4 (high, more than 30 oocysts per sample).

A McMaster count was performed to quantify the strongylid eggs per gram of faeces (EPG). For this, 4 g of faeces was homogenized in approximately 15 ml of saturated sodium chloride solution and sieved into a measuring cylinder. The flotation solution was added to a final volume of 60 ml; the suspension was mixed thoroughly in a stirring flask and immediately transferred to two McMaster counting chambers (150 µl each) and left for 5 min before examination at 100× magnification. The detection limit for EPG was 50. All animals with 150 EPG and higher were included in a faecal egg count reduction test (FECRT). The owners were informed and further instructions on treatment and the second faecal sampling were provided.

According to the results of the questionnaire, alpaca owners predominantly used monepantel, moxidectin or fenbendazole as anthelmintics. The decision on which drug was to be used in the study was based on which drugs had been recently used in the herd (this was then used for the FECRT). Monepantel (Zolvix^®^, Elanco, Bad Homburg, Germany; 7.5 mg/kg p.o.), moxidectin (Cydectin^®^, Zoetis, Parsippany, NJ, USA; 0.4 mg/kg p.o.) or fenbendazole (Panacur^®^, MSD/Intervet, Schwabenheim, Germany; 10 mg/kg p.o.) were applied according to dose recommendations for South American camelids [5, 15–17]. The animals were dosed individually based on body weight (determined by weighing or estimated by the attending veterinarian).

Individual faecal samples were collected after treatment, transferred to the laboratory and examined by McMaster counting to calculate the reduction in egg excretion.

### Larval cultivation

Faecal samples collected before treatment were pooled by farm (20–50 g), homogenized in water and mixed with vermiculite to achieve a moist crumbly structure. The samples were kept at 25 °C for seven to ten days under daily aeration and moistening. After that, third-stage larvae were collected and differentiated microscopically (40× magnification) after staining with Lugol’s solution.

### Statistical evaluation

Descriptive statistics were carried out in Microsoft Excel. For calculation of the FECR and corresponding confidence intervals the program eggCounts-2.1-1’ in R (v.3.5.0) was used [18]. Calculations were done with the standard “two sampled paired” model and the “model with individual efficacy” [19].

## Results

### Sample procurement and faecal consistency

Overall, 617 samples [mean of 22.9 samples/herd, standard deviation (SD) = 11.7] from 538 animals (mean 21.1 animals/herd; SD = 9.2) were examined from April to November 2018. On farms with less than 20 alpacas, animals (*n* = 34) were repeatedly enrolled to receive a sufficient number of samplings per farm. Twelve alpacas did not respond to treatment (FECR < 70% after first treatment) and were treated again with a different anthelmintic. In both cases of repeated treatments, EPG values after the previous treatment were used as pre-treatment values in the FECRT.

Faecal consistency was determined for 590 samples. Of these, 50.5% were of physiological consistency (score 1), 29.3% had soft formed faeces (score 2), 19.0% were soft (score 3) and 1.7% were semi-liquid (score 4).

### Qualitative results of coproscopy

**Fig. 1:**
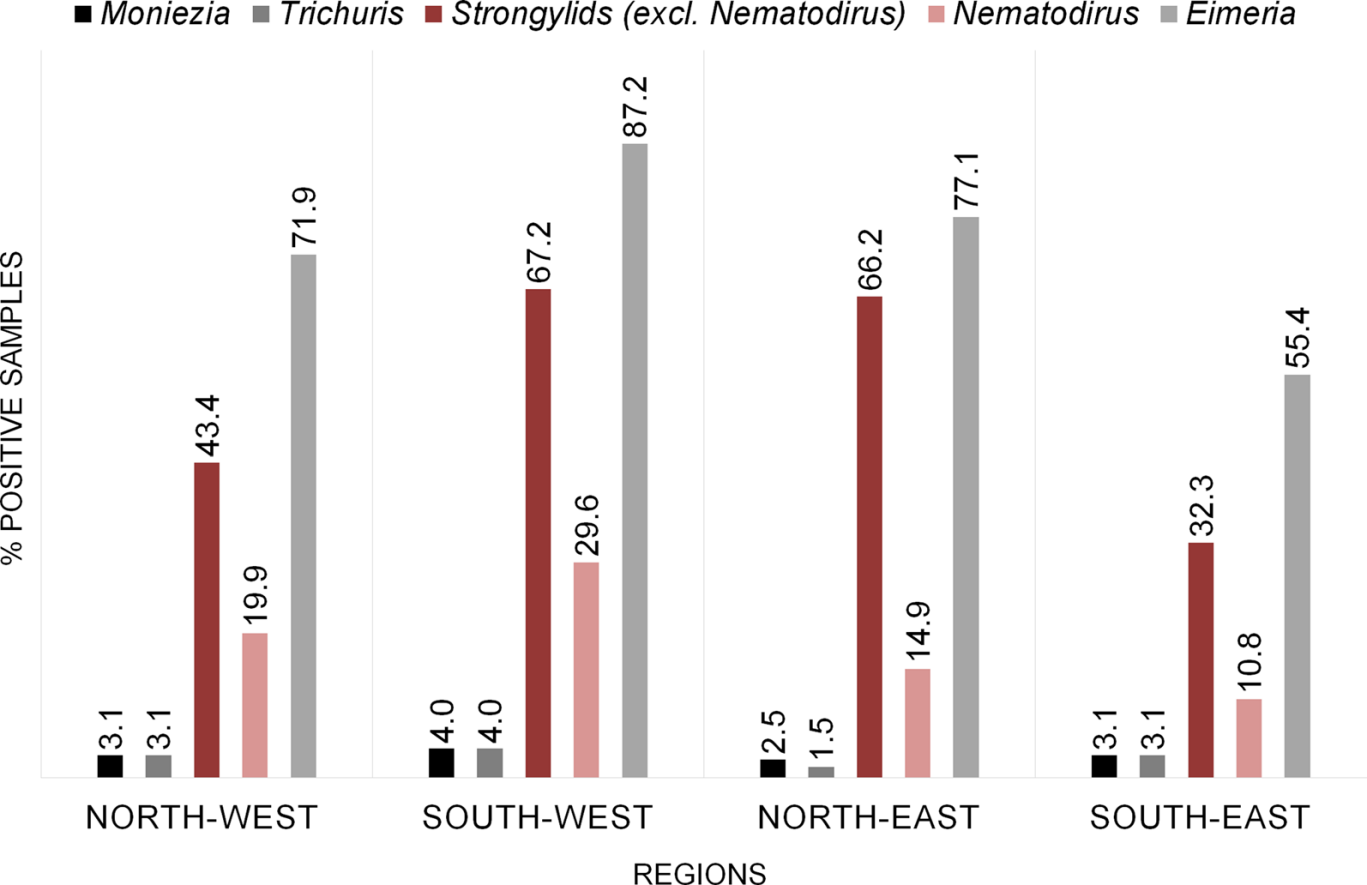
Geographical distribution of prevalence rates for the endoparasites detected by flotation (*n* = 587 samples; see Table 1 for details)

A total of 587 samples were examined both by flotation and the McMaster technique. For 24 of these, the amount of faeces was below than the minimum required for flotation. For an additional 30 samples, only McMaster results were obtained (617 samples were examined by flotation and/or McMaster counting). The most frequently detected parasite stages were oocysts of *Eimeria* spp. (441/587 samples; 75.1%). Of these 76.9% showed a very low grade, 16.3% a low grade, 5.7% a medium grade and 1.1% a high grade of excretion. Of the helminths, strongylids were most common: 55.0% of the 587 samples examined by flotation were positive. Out of the 617 samples examined either by flotation or McMaster or both, 373 (60.5%) were positive for strongylid eggs by at least one method: 41.8% were positive in both; 15.6% were positive in flotation but negative by McMaster counting; and 3.1% were negative by flotation and positive by McMaster. *Nematodirus* eggs were detected in 19.3% of the samples, cestode eggs in 3.1% and *Trichuris* eggs in 2.7% of the samples examined by flotation. Regarding the geographical distribution of the different parasites in the samples, minor variations in prevalence rates could be seen but the parasites detected were found in all regions of Germany (Fig. 1).

In the 587 samples examined by flotation, no correlation between faecal score/diarrhoea and excretion of strongylid eggs or coccidia oocysts could be determined (details not shown). Two of the 27 farms enrolled did not provide samples positive for strongylids, and larval cultures from another two farms with positive samples in flotation did not yield larvae. In the remaining 23 pooled farm samples, *Haemonchus* was detected most frequently (Table 2).

**Table 2:** Results of larval cultures from pooled faecal samples by farm (*n* = 23 pooled samples which yielded positive results)

Detected strongylid genera and mixed infections	% positive farms
Strongylid genus present	
*Haemonchus*	87.0
*Cooperia*	43.5
*Trichostrongylus*	21.7
*Ostertagia*	13.0
*Nematodirus*	4.3
*Oesophagostomum*	4.3
Number of genera present	
1	43.5
2	30.4
3	17.4
4	4.3

### Faecal egg count reduction test

A total of 617 pre-treatment samples were examined by McMaster counting, and 49.9% were positive. The mean EPG (± standard deviation, SD) of all samples was 114.0 ± 251.5, with a maximum of 2650 EPG. In 215 samples (34.8%) EPG values were 150 or higher, and in 208 of the latter cases, treatment was carried out and a second sample could be obtained. The mean body weight (± SD) of these animals was 58.6 ± 13.8 kg (median, 60.0 kg; minimum, 21.9 kg; maximum, 100.0 kg), and animals were dosed by weight. The average number of days between treatment and the second sampling was 16.3 ± 1.6 (minimum, 13 days; maximum, 25 days). Between 66 and 71 samples were examined after treatment with either fenbendazole, moxidectin or monepantel. Overall, 67.5% of the samples were negative in the McMaster examination after treatment (Table 3), most of which were in the moxidectin and monepantel treated groups.

**Table 3:** Results of the faecal egg count reduction test (FECRT)

	Fenbendazole	Moxidectin	Monepantel
No. of herds^a^	12	12	10
No. of samples	71	71	66
Average EPG before treatment ± SD	324.6 ± 387.6	312.7 ± 378.0	280.3 ± 311.1
Average EPG after treatment ± SD	178.2 ± 359.7	23.9 ± 103.1	11.4 ± 43.7
% of samples with EPG = 0 after treatment	26.8	87.3	90.9
No. of farms with EPG > 0 (No. of farms with > 10% of samples with EPG > 0)	12 (8)	6 (4)	2 (0)
FECR in % (95% uncertainty interval)	45 (35–52)	92 (89–95)	96 (93–98)
FECR in % (individual efficacy) (95% uncertainty interval)	62 (48–73)	100 (99–100)	100 (98–100)

^a^Sum > 27, since some farms used different compounds

*Abbreviation*: SD, standard deviation

Repeated treatment with change of the anthelmintic drug was conducted in 25 animals; one of them received all three different drugs consecutively due to insufficient FECR (see below). At the first treatment, 22 of these animals received fenbendazole and three moxidectin. The animals treated with fenbendazole as a first treatment received either moxidectin (*n* = 18) or monepantel (*n* = 4) as a second treatment. Moxidectin application resulted in 17 negative results. One animal still had a persisting EPG of 800, was re-treated with monepantel and became negative after that. Monepantel as a second treatment resulted in 7/8 negative results and one with an EPG of 150. The animals that were still positive after moxidectin as a first treatment all received monepantel. Two weeks later, two of them had a negative faecal egg count and one had an EPG of 150.

When all animals in each treatment group were combined and analysed in the two samples paired model, the fenbendazole-treated group displayed an average FECR of 45%. For the moxidectin-treated group the calculated FECR was 92%, and in the monepantel-treated group 96%. When the model was applied taking individual efficacy into consideration, fenbendazole treatment had an efficacy of 62%, moxidectin and monepantel of 100% (Table 3).

**Fig. 2:**
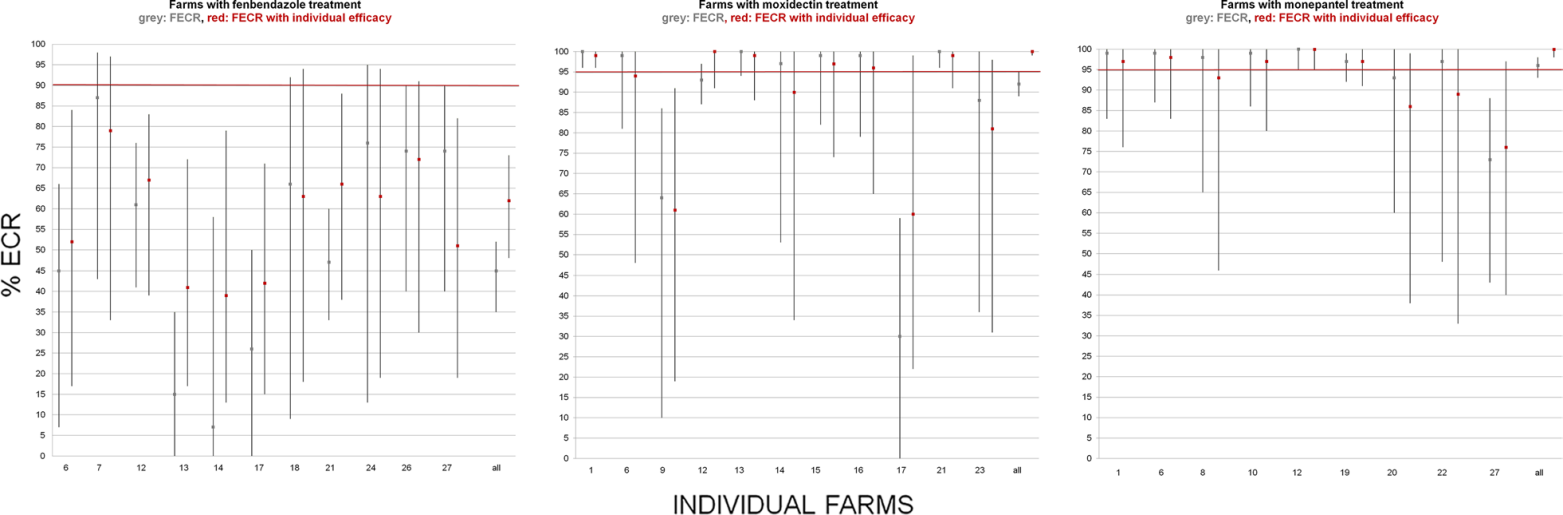
Faecal egg count reductions (mean and confidence intervals) for individual farms for three different anthelmintic drugs used. Horizontal red lines show the expected level of susceptibility

At the herd level, the farms which used fenbendazole showed treatment efficacies of 15–87% (41–79% when individual efficacy was considered). The farms under moxidectin treatment had FECR rates of 30–100% (60–100% with individual efficacy consideration) and the farms with monepantel treatment showed 73–99% FECR (86–99% with individual efficacy consideration) (Fig. 2).

**Fig. 3:**
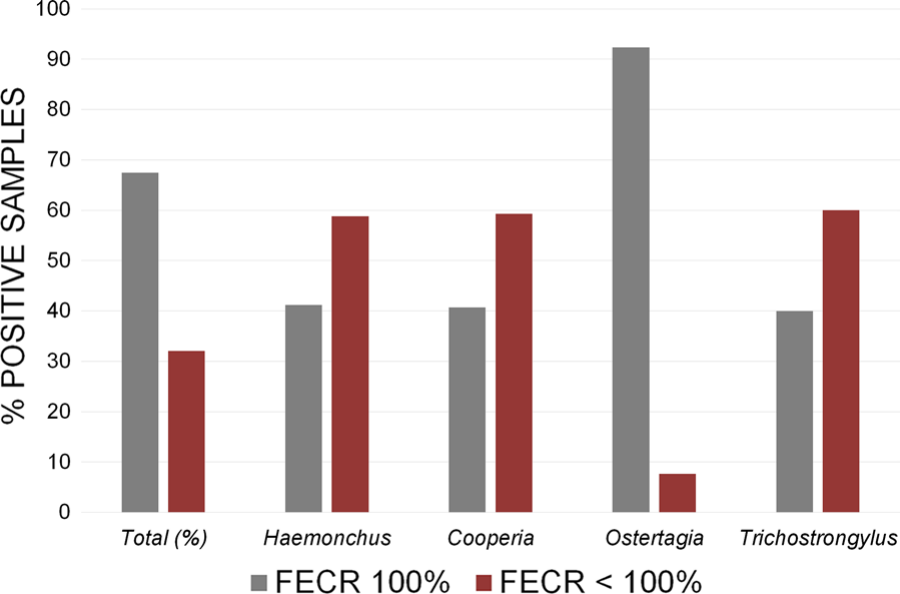
Relative composition of strongylid genera before treatment in relation to samples with faecal egg count reduction (FECR) of 100% *vs* FECR of < 100%

*Haemonchus* larvae were found in pre-treatment samples on all farms (*n* = 9) with poor FECR (defined as farms with a FECR < 100% in > 10% of the samples). *Cooperia* larvae were detected on six of these farms, *Trichostrongylus* on four; all three genera were overrepresented on these farms (Fig. 3). Farms with a poor FECR were found in all areas of Germany (north-west: 3/9, south-west: 2/6, north-east: 4/9 farms) except the south-east (Bavaria; 3 farms).

## Discussion

### Coproscopical results

Endoparasitoses are a common problem in the South American camelids. Losses in alpacas in Peru due to nematode infections of the gastrointestinal tract are estimated to be 46.3% of the total losses [20]. In the present study, coccidia (genus *Eimeria*) were the most common endoparasites detected. In South American camelids, five different *Eimeria* species (*E. alpacae*, *E. lamae*, *E. punoensis*, *E. macusaniensis* and rarely *E. ivitaenis*) can be found [5, 21]; *E. peruviana* is currently not validated as a species. Especially *E. macusaniensis* is frequently implemented in clinical cases [21–23]. Due to the high host specificity *Eimeria* spp. are not transmitted between South American camelids and other species [21, 22]. As we examined only animals older than one year we found mostly very low or low excretion densities; however, in some cases animals shed considerable amounts of oocysts, indicating that coccidiosis may occur in alpacas under unfavourable conditions on the farm or might be facilitated by a poor immune status of the animals.

In South American camelids different gastrointestinal strongylids can be found [5, 24–26]. In addition to host-specific species, South American camelids can also be infected with strongylid species of Old World camelids (*Camelostrongylus mentulatus*) as well as species of wild and domestic ruminants [5]. Of the camelid-specific nematodes, *Spiculopteragia peruvians* is so far only known to occur in South America [5] and *Graphinema aucheniae* in South America and Australia [26] while *Lamanema chavezi* has also been detected in llamas in New Zealand [27] and the USA [28]. The latter is rather pathogenic to the camelid host as its enterohepatic migration induces hemorrhagic enteritis and hepatitis [29]. There is no indication of its occurrence so far in Europe; however, *Lamanema* eggs are similar to those of *Nematodirus* and can only reliably be differentiated by molecular tools [30]. Both can easily be missed in flotation due to their high density [31], so special attention must be paid on this strongylid species. Recently, the first infection with the camelid-specific *Nematodirus lamae* in Europe was reported from the UK in a case of sudden death of an alpaca [32] and the authors pointed out that moving of animals is of high importance in spreading parasites to new areas. The genus *Nematodirus* is commonly reported from both domesticated and wild South American camelids. Besides *N. lamae*, species infecting ruminants (*Nematodirus abnormalis*, *N. battus*, *N. filicollis*, *N. helvetianus*, *N. spathiger*) can also be found in South American camelids [5, 26, 33–36]. *Nematodirus* was detected in 19.3% of the alpaca samples, making this genus the third-most common parasite in the examined population. The eggs of *Nematodirus* are easily differentiated from those of the families Trichostrongylidae or Chabertiidae and were not considered in the faecal egg count reduction test. The ruminant strongylids *Ostertagia ostertagi*, *O. lyrata*, *Teladorsagia circumcincta*, *Marshallagia marshalli*, *Haemonchus contortus*, *Trichostrongylus axei*, *T. colubriformis*, *T. longispicularis*, *T. vitrinus*, *Cooperia oncophora*, *C. pectinata*, *C. punctata*, *Bunostomum trigonophorum*, *Oesophagostomum columbianum*, *O. venulosum* and *Chabertia ovina* can also infect South American camelids [5, 7, 11, 25, 26, 35–39]. Depending on the worm burden, the nematode species involved and the age and constitution of the host animal, nematode infection can lead to different degrees of disease progression, from subclinical and mild disease with unspecific signs, like decreased appetite, emaciation and dull coat, to severe cases with diarrhoea, apathy, hypoalbuminemia and anaemia [6].

Of the strongylids transmitted from small ruminants, *H. contortus* probably plays the most significant role in the South American camelids and accounts for the greatest losses due to endoparasitoses [40]. A recent study from Australia confirmed the high infection rates and worm burdens for *H. contortus* in alpacas [26].

Whipworms (*Trichuris barbetonensis*, *T. cameli*, *T. discolor*, *T. globulosa*, *T. ovis*, *T. skrjabini*, *T. tenuis*, *T. tumens*) are relatively common parasites of South American camelids as well as ruminants [25, 26, 39] as re-infections with the long-lived and highly resistant larvated eggs are common [23]. *Trichuris* eggs were found in only 5.7% of the samples, which may be an underestimate, as these eggs require a higher density of flotation solution to reliably be detected in flotation. This must also be assumed for the large *Nematodirus* eggs and the thick-shelled oocysts of *Eimeria macusaniensis* [41].

Tapeworms of three genera can occur in alpacas: *Moniezia* (*M. expansa*, *M. benedeni*), *Thysaniezia* (*T. ovilla*) and *Thysanosoma* (*T. actinioides*) [20, 23, 38, 42–48]. *Moniezia* infections have been reported in South American camelids from central Germany [23] and identified as *M. expansa* and *M. benedeni* in Switzerland [49]. Although none of the used drugs in this study is effective against cestodes in alpacas (except fenbendazole which requires doses of 50 mg/kg BW; [5]), the infection rate in the present study was low (3.1%) and these parasites seem to be generally rare in South American camelids [23], so little is known about their pathogenicity.

### Faecal egg count reduction test

Only animals with a pre-treatment faecal egg count of 150 EPG or higher were included in the treatment. South American camelids generally appear to shed relatively low numbers of gastrointestinal nematode eggs, which may be due to a lower infection pressure as these animals do not spread their faeces across pasture but deposit them in specific places (“latrines”; [33, 50]). In addition, they seem to be less susceptible to the nematodes of small ruminants and consequently excrete fewer eggs [51, 52].

In the case of fenbendazole, 73.2% of the animals still excreted eggs after treatment. After treatment with moxidectin, eggs could still be detected in the faeces of 12.7% of the samples, and after monepantel treatment, 9.1% of the animals were still positive upon the second faecal examination. Fenbendazole had by far the lowest efficacy. Even when an efficacy of only 90% is expected [53], only one additional sample and none of the farms met this cut-off. For moxidectin and monepantel, efficacy was generally high but single animals and farms clearly showed persistent egg excretion. It is unclear whether this is sign of anthelmintic resistance of the worms involved or due to poor bioavailability of these drugs in alpacas [6, 54]. In a previous study in the USA, fenbendazole was used to treat alpacas with 10 mg/kg of body weight and the FECRT revealed a complete lack of efficacy [55]. The authors concluded that this was due to resistance; however, no confirmatory *in vitro* study was carried out. Additional tests have to be conducted to unambiguously determine anthelmintic resistance, such as the composition of strongylid genera post treatment (unfortunately, this could not be established in the present study due to technical constraints) and assays for further evaluation of susceptibility of the nematodes to the applied drugs [55]. The recommended doses for small ruminants were previously simply transferred to the South American camelids. However, the latter can display very different pharmacokinetic drug profiles, e.g. for fenbendazole two to four times the doses for sheep are recommended [16, 17], and for monepantel three times the dose recommended for sheep is necessary to achieve a sufficient egg count reduction [15]. Underdosing of anthelmintics not only leads to poor reduction of the worm burden, it can also drive the development of resistance in camelid nematodes [6]. If this is the case on alpaca farms, the emergence of anthelmintic resistance (especially against fenbendazole) is currently significantly promoted. Underdosing due to body weight underestimation (which can largely be excluded in the present study since weighing scales were used in a number of farms and one of the authors, LK, provided assistance in correct weight determination) or due to incomplete swallowing of the drug (which South American camelids are prone to because of spitting) can contribute to this problem on the farms.

Infections with gastrointestinal strongylids mostly take place on pasture and many species can undergo hypobiosis in the mucosal layer of the intestinal wall in the winter months to be reactivated in spring, so that during this time, anthelmintic treatment is frequently unsuccessful [33]. To rule out this source of error for this study, the investigations took place in the period from April to October.

The FECRT is difficult to interpret in terms of anthelmintic resistance since egg excretion was low in most cases and studies evaluating the actual efficacy of different doses of anthelmintic drugs in alpacas (including determination of effective doses by titration and determination of dose-limiting parasite species; see [55, 56]) are not available. However, the high number of treatment failures especially after fenbendazole application (24/71 samples had a FECR of < 50% after treatment) *versus* complete cessation of egg excretion (19/71 samples had a FECR of 100%) indicates considerable variations in the susceptibility of the different strongylid populations to treatment with different anthelmintics. This reflects the owners’ concern noticed in the questionnaire survey conducted previously [4] where 15% of the owners suspected unsatisfactory efficacy of anthelmintic treatment. However, there was only an incomplete alignment of the owners’ perception with the results of the test; of the 17 farms which had FECR results < 100%, only six (35.3%) reported poor efficacy or losses due to endoparasites, while one of the ten farms with complete FECR had indicated reduced efficacy in the questionnaire [4].

Taking geographical differences or farm structures into consideration, cases of insufficient FECR were found all over Germany. In general, the investigated herds were all medium to large as defined previously [4] with a minimum of 11 alpacas per farm, but on small farms the numbers of available samples were limited, so data from these farms need to be interpreted with caution. A FECR < 100% was seen in one or two samples/farm in ten cases (three of them from farms with a limited sample size); on six farms with a FECR < 100% the percentage of affected samples ranged from 16.9 to 37.5% (5–11 samples/farm) and involved treatment with all three drugs overall. Since the used dosages were adapted to alpacas [5, 15–17] and were sufficient to reduce egg excretion in 26.8, 87.3 and 90.9% of the animals after fenbendazole, moxidectin or monepantel treatment, respectively, general underdosing is unlikely. Although more detailed studies will be required to unequivocally determine the presence and extent of resistance of the investigated nematode populations against the applied anthelmintic compounds, indirect evidence supports the assumption that the efficacy of the anthelmintic treatment applied in this study may already be compromised in the examined alpaca herds. The larval cultures revealed the presence of nematodes of small ruminant origin, and for these strongylids resistance against benzimidazoles, levamisole or moxidectin has been shown in a number of studies from Germany [57–62] and neighbouring countries such as Switzerland [49, 61, 63], Austria [64, 65], Belgium [14] and the Netherlands [66]. Furthermore, in the UK monepantel resistance has lately been reported [67], so it must be assumed that transmission of strongylids from small ruminants to South American camelids may also include resistant populations. In Peru, anthelmintic resistance to benzimidazoles and macrocyclic lactones has been reported in trichostrongylids of alpacas [68, 69], and in Australia, resistance in strongylids (primarily *Haemonchus*, *Trichostrongylus*, *Camelostrongylus*, *Ostertagia* and *Cooperia*) of alpacas against ivermectin, fenbendazole, closantel and moxidectin has been described [13].

In addition, in most studies on anthelmintic resistance, *H. contortus* has been implicated as a major driver (e.g. [11, 14]), and this nematode was the most abundant in the larval cultures examined in this study, although it was previously considered to be less frequent in New Wold camelids than in small ruminants [44, 50, 70]. We confirm that *H. contortus* is a common strongylid species of South American camelids, and we further hypothesize that this high representation of the Barber’s Pole worm in the examined samples was promoted by the presence of resistant worms of this species. To a lesser extent this also applies to *Cooperia* and *Trichostrongylus* which have also been inferred in anthelmintic resistance in camelids [11, 12, 14, 68]. Since *H. contortus* is not only pathogenic in small ruminants but also in South American camelids [8, 26, 40], the presence of this species and its response to anthelmintic treatment should be monitored thoroughly, especially in view of cross-transmission between ruminants and alpacas as described to be considerable in recent studies from Australia [26]. Co-grazing could not be identified as a risk factor in the present study since it was not practiced on any of the examined farms (although on one farm after-use of pasture by goats was reported; this farm was not included in the FECRT due to low/negative McMaster results). In addition, *C. oncophora* is the dose-limiting species in anthelmintic efficacy in cattle [71] so this species could benefit from the low doses of anthelmintic applied.

## Conclusions

The present study confirms that gastrointestinal strongylids are common parasites in German alpaca herds and animals frequently excrete eggs of these nematodes, although mostly in lower amounts compared to small ruminants. Only two of the 27 examined farms provided samples negative for gastrointestinal strongylids. *Haemonchus* was the most prevalent genus, indicating that previous anthelmintic treatments may have been insufficient to eliminate it effectively. Fenbendazole was not sufficiently effective at the dose recommended for South American camelids, and the possibility of resistance development cannot be ruled out. Although moxidectin and monepantel had an overall satisfactory efficacy, it was reduced on some farms. Again, a possible establishment of resistant parasites transferred from small ruminants cannot be excluded. Despite the limitations of the FECR for samples with generally low egg excretion, the results highlight the importance of faecal examinations for monitoring of the infection status of the herd and an indication of treatment necessity and success. In addition, correct dosing is mandatory to maintain treatment success and efficacy. Frequent deworming of all animals in a herd must be considered obsolete as it promotes resistance through strong selection pressure [71, 72]. In line with other domestic animal species, adequate control of strongylid infections in South American camelids requires monitoring of faecal egg shedding, selective (ideally, targeted selective) treatment and post-treatment coproscopical examination to evaluate treatment success, as well as clinical observations of the animals for signs of parasitic disease.

## Data Availability

All data generated or analysed during this study are included in this published article.

## Abbreviations

FECRT: faecal egg count reduction test
EPG: eggs per gram of faeces
SD: standard deviation
